# Accuracy of Plasmodium falciparum genetic data for estimating parasite prevalence and malaria incidence in Uganda

**DOI:** 10.21203/rs.3.rs-8251834/v1

**Published:** 2025-12-09

**Authors:** Shahiid Kiyaga, Monica Mbabazi, Thomas Katairo, Kisakye Diana Kabbale, Victor Asua, Bienvenu Nsengimaana, Innocent Wiringilimaana, Francis Ddumba. Semakuba, Caroline Mwubaha, Jackie Nakasaanya, Eric Watyekele, Alisen Ayitewala, Stephen Tukwasibwe, Jerry Mulondo, Samuel Lubwama. Nsobya, Bosco Agaba, Catherine Maiteki-Sebuguzi, Moses Robert. Kamya, David Patrick. Kateete, Joyce Nakatumba Nabende, Daudi Jjingo, Gerald Mboowa, Charles Batte, Isaac Ssewanyana, Andrés Aranda-Díaz, Grant Dorsey, Philip J. Rosenthal, Melissa Conrad, Bryan Greenhouse, Jessica Briggs

**Affiliations:** Makerere University; Makerere University; Infectious Diseases Research Collaboration.; Makerere University; Infectious Diseases Research Collaboration.; Infectious Diseases Research Collaboration.; Central Public Health Laboratories; Infectious Diseases Research Collaboration.; Infectious Diseases Research Collaboration.; Infectious Diseases Research Collaboration.; Infectious Diseases Research Collaboration.; Central Public Health Laboratories; Infectious Diseases Research Collaboration.; Infectious Diseases Research Collaboration.; Infectious Diseases Research Collaboration.; National Malaria Elimination Division, Ministry of Health; National Malaria Elimination Division, Ministry of Health; Makerere University; Makerere University; Makerere University; Makerere University; Broad Institute of MIT and Harvard; Makerere University; Central Public Health Laboratories; ISGlobal; Division of HIV, Infectious Diseases and Global Medicine, Department of Medicine, University of California San Francisco; Division of HIV, Infectious Diseases and Global Medicine, Department of Medicine, University of California San Francisco; Division of HIV, Infectious Diseases and Global Medicine, Department of Medicine, University of California San Francisco; Division of HIV, Infectious Diseases and Global Medicine, Department of Medicine, University of California San Francisco; Division of HIV, Infectious Diseases and Global Medicine, Department of Medicine, University of California San Francisco

**Keywords:** Malaria, Uganda, Plasmodium falciparum, Molecular surveillance, Complexity of infection, Parasite diversity, Genomic epidemiology

## Abstract

**Background:**

Genetic metrics derived from *Plasmodium falciparum* infections offer a potential complement to conventional malaria surveillance by utilizing features of parasite diversity and relatedness to estimate transmission intensity. However, the performance of molecular metrics to predict epidemiologic metrics across a wide range of transmission settings remains understudied.

**Methods:**

Dried blood spots from 3563 symptomatic malaria cases were collected from 26 sentinel health facilities across Uganda during two collections in 2023. Amplicon deep sequencing of 165 polyallelic microhaplotypes was performed using MAD^4^HatTeR. Within-host diversity metrics (complexity of infection (COI), effective complexity of infection (eCOI), percent polyclonality, within-host relatedness) and between-host relatedness metrics were calculated. Associations with prevalence and recent incidence were evaluated using correlation and regression analyses, and estimation accuracy was examined using nested grouped cross-validation.

**Results:**

Marked geographic heterogeneity in malaria burden was evident across sites; parasite prevalence ranged from 5.0% to 49.23% in Round 1, while incidence ranged from 91–1062 cases per 1,000 person-years (PY) in Round 1 and 33–1667 cases per 1,000 PY in Round 2. COI and eCOI were strongly and consistently positively associated with parasite prevalence. The proportion of highly related infection pairs was negatively associated with both prevalence and incidence and was the genetic metric most consistently associated with incidence. Nested grouped cross-validation identified single-predictor models using COI or eCOI as optimal for estimating prevalence, yielding a pooled cross-validated correlation of *r* = 0.79. Models estimating incidence showed weaker performance, with models incorporating both diversity and relatedness metrics achieving a pooled correlation of *r* = 0.37.

**Conclusions:**

Microhaplotype-based metrics of within-host diversity, particularly COI and eCOI, reliably reflected spatial variation in malaria prevalence across Uganda, while between-host relatedness provided complementary information and was the strongest predictor of incidence. These findings indicate that parasite genomic metrics derived from polyallelic microhaplotypes can capture broad differences in transmission intensity reflected by parasite prevalence, but may have more limited ability to predict incidence. Integration of genomic metrics with harmonized epidemiologic data and expanded sampling of asymptomatic infections will be important next steps to understand the potential utility of parasite genetic metrics for malaria surveillance and subnational stratification.

## Introduction

Despite sustained investments in malaria control, malaria due to *Plasmodium falciparum* remains a major public health burden in Uganda, which has the 3rd highest case rate globally (1). While interventions such as long-lasting insecticide-treated nets (LLINs), indoor residual spraying, and artemisinin-based combination therapies have contributed to substantial reductions in morbidity and mortality, progress has stalled and malaria transmission remains high in much of the country (1, 2). In recent years, there has been growing programmatic use of malaria molecular surveillance (MMS) to complement traditional surveillance systems and support national malaria control programs (NMCPs) with real-time, actionable data (3–5). To date, MMS has primarily been used to track well-characterized mutations associated with drug and diagnostic resistance (6–8). Beyond this clear use case, a major question is whether parasite genomic data can be used to infer transmission intensity to inform malaria control strategies. This remains a methodological challenge as highlighted by the WHO’s 2019 position paper on the use of parasite genetics in malaria surveillance (9). Phylodynamic methods used to understand the transmission histories of viral pathogens from molecular data are inappropriate for *P. falciparum*, which undergoes sexual reproduction within the mosquito vector before transmission to a new human host and experiences transmission bottlenecks between both the mosquito and human hosts (10). Additional factors such as varied host immunity and co-transmission of multiple parasite strains from one mosquito bite, further complicate the relationship between the genetic diversity of the parasite population and within-host infections (10, 11). Despite these obstacles, genomic metrics have shown promise for inferring transmission intensity in both empiric and modelling studies.

The complexity of infection (COI), defined as the number of genetically distinct parasite strains within an individual host, has been associated with transmission intensity in several studies, with higher COI generally observed in higher transmission settings due to frequent superinfection (parasite strains acquired over multiple mosquito bites) (12–14). The proportion of polyclonal infections (COI > 1) has likewise been correlated with transmission intensity (15). Modeling studies mirror these empirical findings, demonstrating that COI-based metrics show strong correlations with malaria prevalence, and that superinfection dominates in high-transmission settings (10, 16, 17). In contrast to COI, which is most informative in moderate to high transmission settings, modeling studies predict that identity-by-descent (IBD)-based metrics may be valuable in low transmission settings where reduced effective population size and limited out-crossing result in more highly related parasites (18). IBD analyses have successfully characterized fine-scale spatial connectivity, selection signatures, and decreases in transmission in studies in the Thailand-Myanmar border (19), South America (18), and Senegal (13).

Despite growing interest in using parasite genetics for malaria surveillance, several methodological limitations constrain current approaches. Some genotyping methods—including SNP barcoding, which can assess only limited diversity in the setting of polyclonal infections, and those with limited sensitivity for minority clones (15), fail to fully characterize the allelic diversity in complex infections necessary for accurate COI estimation (20–24). Additionally, studies have relied on epidemiologic metrics of varied quality for comparison with parasite genomic metrics, largely driven by differences in local data availability and surveillance infrastructure. Parasite prevalence, while commonly available, has a complex, non-linear relationship with transmission intensity that varies by endemicity level, population immunity, and sensitivity of the diagnostic used (25). Incidence data are more directly related to current transmission intensity but typically derived from routine Health Management Information Systems (HMIS) that suffer from incomplete case capture, lack of age or clinical stratification, and inclusion of non-parasitologically confirmed diagnoses (26, 27). Consequently, establishing the quantitative relationship between both within-host and population-level parasite genetic diversity and transmission intensity, validating methods to predict epidemiologic metrics from genomic data, and demonstrating the utility of genetics for monitoring intervention impacts remain research priorities (9, 11). However, only one other study, in Senegal, has attempted to predict incidence from parasite genomic metrics (15).

To address this research priority, we used samples collected from 26 sites across Uganda as part of the Implementing Malaria MoleculaR SurveillancE in Uganda (IMMRSE-U) study, a nationwide MMS initiative integrated with a network of sentinel surveillance sites with enhanced incidence data (28). We generated amplicon deep sequencing data from diverse polyallelic microhaplotype markers in the sensitive MAD4HatTeR amplicon sequencing panel (29) and applied computational methods designed for polyallelic data to estimate metrics of within-host parasite diversity and between-host relatedness. This study aimed to assess the ability of parasite genetic metrics to accurately estimate site-level prevalence and incidence across a range of transmission intensities in Uganda.

## Methods

### Study design

The IMMRSE-U parent study is a malaria molecular surveillance study that collected dried blood spots (DBS) from 200 patients with uncomplicated malaria twice yearly at 30 sites across Uganda. For this analysis, we included samples collected from 26 sites with incidence data available from the Uganda Malaria Surveillance Project (UMSP) managed by the National Malaria Elimination Division (NMED), Ministry of Health and the Infectious Disease Research Collaboration (IDRC). This study includes samples from two collections in 2023: Round 1 – dry, lower transmission season (January–March 2023) and Round 2 – wet, higher transmission season (July–September 2023) (30).

### Epidemiological data

Malaria incidence data were extracted from the UMSP database at the site level and calculated for six time periods: 3 months, 6 months, and 12 months prior to start of Round 1 and Round 2 sample collection. The primary outcome was malaria incidence rate in the 3 months prior to sample collection.

For Spearman’s rank correlation tests and visualization, we used geometric mean malaria incidence per 1000 person-years in the last 3 months. Site-level geometric mean incidence per 1000 person years in the last 3 months was categorized into five strata: very low (0–150), low (150–300), moderate (300–450), high (450–600), and very high (≥ 600) for mapping. For statistical modeling, we used raw counts with denominators as offsets to account for differences in population size.

Prevalence data were collected via cross-sectional surveys conducted as part of a randomized controlled trial of (LLINs) of children aged 2–10 years in the target areas surrounding 17 of the IMMRSE-U health facilities from November 2022-March 2023 (31), which aligned with the timing of the Round 1 sample collection. Prevalence was defined as the proportion of children aged 2–10 years with microscopy-confirmed parasitemia and categorized as very low (0–10%), low (10–20%), moderate (20–30%), high (30–40%), and very high (> 40%) for mapping.

### Laboratory methods

Genomic DNA was extracted from dried blood spots (DBS) using an established Tween-20/Chelex-100 method (32). Parasite density was determined using the *var*ATS quantitative PCR (qPCR) assay targeting multicopy subtelomeric sequences of *Plasmodium falciparum* (33). The resulting parasite density estimates guided sample selection for downstream genotyping, with the goal of sequencing 100 samples per site. For sites with > 100 samples having parasite densities > 1,000 parasites/μL, a total of 100 samples at these densities were selected at random. For sites with fewer than 100 samples > 1,000 parasites/μL, additional lower-density samples were included until a total of 100 was reached.

Amplicon libraries were prepared using the Multiplex Amplicons for Drug, Diagnostic, Diversity, and Differentiation Haplotypes using Targeted Resequencing (MAD^4^HatTeR) protocol as previously described (29) using primer pools D1.1/R1.2 and R2.1 to capture both high diversity and drug resistance targets. Most relevant for this analysis, pool D1.1 comprises 165 loci with high *P. falciparum* population diversity. Sequencing was performed on the Illumina MiSeq platform.

### Genomic data analysis

Raw reads were processed using the Nextflow-based MAD^4^HatTeR pipeline (29). Samples with ≥ 75% of targeted loci successfully covered at ≥ 100 reads were retained for downstream analysis. Alleles were called when present at a within-sample allele frequency (WSAF) threshold > 1% and covered at ≥ 10 reads (21, 29). Genetic diversity metrics COI, within-host relatedness (WHR), effective COI (eCOI), and allele frequencies were jointly computed using MOIRE v3.5.0 (22), a Bayesian model accounting for genotyping error and within-host relatedness. In this model, eCOI summarizes the effective within-host diversity by adjusting the estimated number of strains according to their genetic relatedness, providing a continuous measure of the number of genetically distinct contributors to each infection. Polyclonal infections were defined as those with eCOI > 1.1. Population-level genetic diversity was assessed by calculating mean heterozygosity (H_e_) for the ten most heterozygous loci across each site. Between-sample relatedness (*r*) was estimated within each site with Dcifer v1.2.1 (20), which accounts for the total proportion of genomes shared between parasites in two infections due to recent common ancestry (IBD). We estimated pairwise relatedness (*r*) between samples and applied two thresholds to classify highly related pairs: *r* > 0 (any detectable relatedness) and *r* > 0.125 (indicating closer genetic relationships). For each site, we calculated the percentage of sample pairs exceeding each relatedness threshold with a p-value < 0.05 after adjustment for multiple comparisons using Benjamini-Hochberg correction.

### Statistical analysis

Statistical analyses were conducted in R version 4.4.3 (34). Parasite density and diversity metrics (COI, eCOI) were compared across age groups (< 5 years, 5–15 years, > 15 years) and between sampling rounds using the Wilcoxon rank-sum test. Site-level associations between genetic metrics and incidence and prevalence were compared using the Spearman rank correlation test and linear regression models.

Nested grouped cross-validation (CV) was used to evaluate generalized linear mixed models (GLMMs) with negative binomial error family estimating site-level malaria incidence; tThe negative binomial family was selected over Poisson based on superior model fit.. Data were analyzed using package lme4. Molecular predictors included COI, eCOI, percent polyclonal infections, percent highly related by IBD, average IBD, H_e_, and WHR; models were limited to one COI metric (COI, eCOI, % polyclonal) and one IBD metric due to collinearity. For single-round analyses, where incidence was estimated separately for sites in Round 1 and Round 2, sites were treated as fixed effects. For the final model combining data across rounds, site was modeled as a random intercept to enable generalization beyond the observed sites while avoiding overfitting and round was included as a fixed effect: *incidence*_*count*_ *~ predictors + round + (1/site) + offset(log(exposure))*. Sites were partitioned into five outer folds, with inner three-fold CV used to select the optimal predictor combination based on root mean squared error (RMSE). Parameter estimation used adaptive optimizers for robust convergence. Model performance was summarized using RMSE, mean absolute error (MAE), and Pearson correlation between observed and predicted incidence. Overall predictive strength was quantified using Fisher’s pooled correlation across repeated CV iterations. Other approaches, including random forests and LASSO regression, were also evaluated within the same nested CV framework; however, these approaches did not outperform the GLMMs, likely due to the limited sample size relative to the number of correlated genetic predictors. An identical nested CV framework was applied to quasibinomial models predicting Round 1 prevalence using the same predictor sets, but only for Round 1 data given that prevalence data were only available at this time point.

## Results

### Study population and sampling

A total of 3563 participants with symptomatic malaria were enrolled from 26 malaria reference centers (MRCs) in Uganda across two rounds of sample collection (Table 1). More samples were collected in Round 2 (July–September) and a higher proportion passed quality thresholds compared to Round 1 (January–March). Prevalence data were only available for Round 1, since only one cross-sectional survey was performed. Incidence data were available for more sites in Round 2 than in Round 1. Median parasitemia was higher in Round 2, which occurred during the higher transmission season. In both rounds, children aged 5–15 contributed the greatest share of samples.

### Heterogeneity in malaria burden

**Figure 1:**
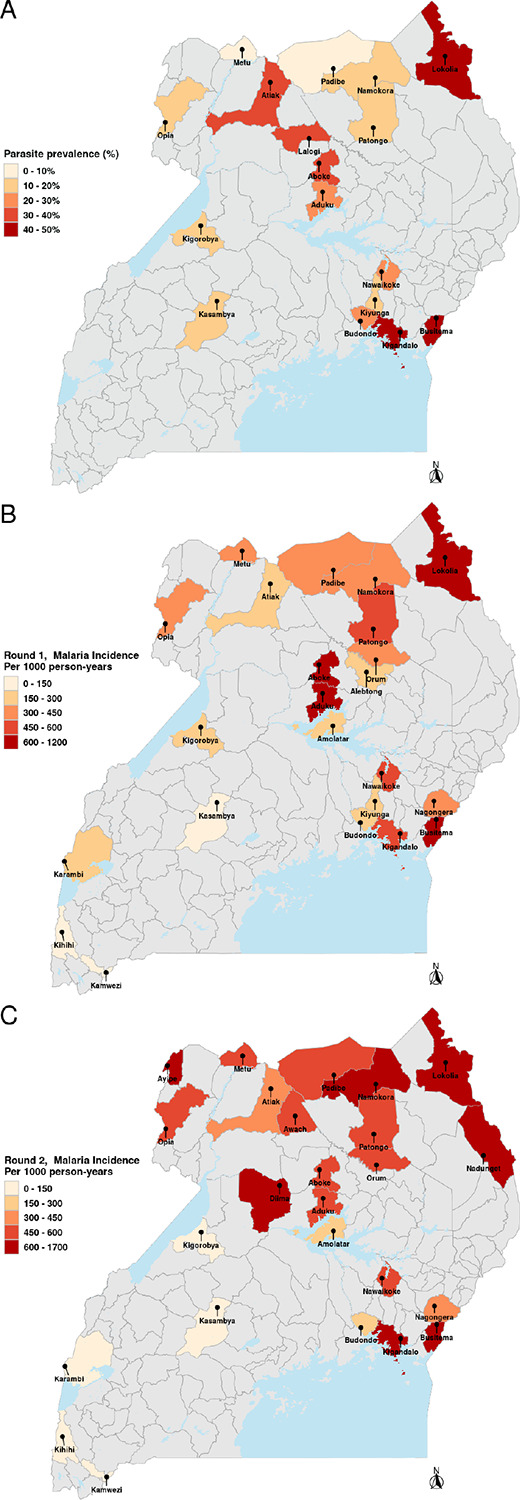
Malaria transmission intensity across MRCs in Uganda. (A) Parasite prevalence (%) from cross-sectional surveys across 17 sites, temporally aligned with Round 1 sample collection, (B) Round 1 incidence (cases per 1000 person-years in the 3 months prior to sample collection) across 23 sites, and (C) Round 2 incidence cases per 1000 person-years in the 3 months prior to sample collection) across 25 sites. Black dots indicate study sites.

Malaria incidence and prevalence varied widely across the study sites, reflecting significant geographic heterogeneity in transmission (Figure 1A–C). Measures consistent with high transmission intensity were consistently observed in northern and eastern Uganda, where some sites recorded incidence rates exceeding 1200 cases per 1000 person-years and parasite prevalence above 40%. In contrast, southwestern regions exhibited markedly lower malaria burden, with incidence rates below 150 cases per 1000 person-years. Parasite density declined with age, as expected (**Supplementary Figure S1A**).

### Heterogeneity in parasite genetic diversity and relatedness

Complexity of infection was high overall, with 74% of participants having polyclonal infections. School-aged children (5–15 years) exhibited the highest COI (mean of 4.2) and eCOI (mean of 3.0) followed by younger children (<5 years) and adults (>15 years; Supplementary Figures S1B-C). Seasonal comparisons revealed higher average parasite density, COI, and eCOI during the peak transmission period (Round 2, July–September) than in the earlier dry season (Round 1, January–March) **(Supplementary Figure S2)**. Other genetic metrics analyzed such as percent polyclonality, H_e_, and percent highly related samples had no significant variations between seasons. These findings indicate that within-host diversity metrics increased during periods of higher transmission, reflecting temporal trends within sites, and that school-aged children exhibited higher within-host diversity than other age groups, likely due to the development of anti-disease more than anti-parasite immunity.

Analysis of site-level average COI revealed strong geographic heterogeneity, with consistently higher values in northern and eastern Uganda, where transmission was highest (maximum COI of 9.0), compared to the southwest, where transmission was lowest (minimum COI of 2.0, **Supplementary Figure S3**). While some sites, such as Namokora and Orum, showed substantial seasonal fluctuation, most sites remained relatively stable. Values for eCOI were consistently lower than COI, reflecting the effect of within-host relatedness **(Supplementary Table 1, Supplementary Figure S4)**. The proportion of polyclonal infections also varied widely, from **45%** to **100%**. Parasite population diversity, assessed through H_e_ at the ten most variable loci, was uniformly high across sites (**0.76–0.86**) **(Supplementary Table 1)**, consistent with the diverse loci assessed and substantial standing genetic variation in *P. falciparum*. Next, we examined the relatedness of infections between participants within each site, determined by the **proportion of highly related pairs (*r* > 0.125)**, accounting for a false discovery rate of 5%. Low transmission sites had the highest percentage of highly related infections (maximum 7.8%), but this metric approached zero in high transmission sites such as Orum and Namokora **(Supplementary Table 1)**. These results show that, as expected, parasite populations in higher burden regions are characterized by higher within-host diversity and lower between-host relatedness than lower burden sites.

### Associations between parasite genetic metrics and transmission intensity at the site level

**Figure 2:**
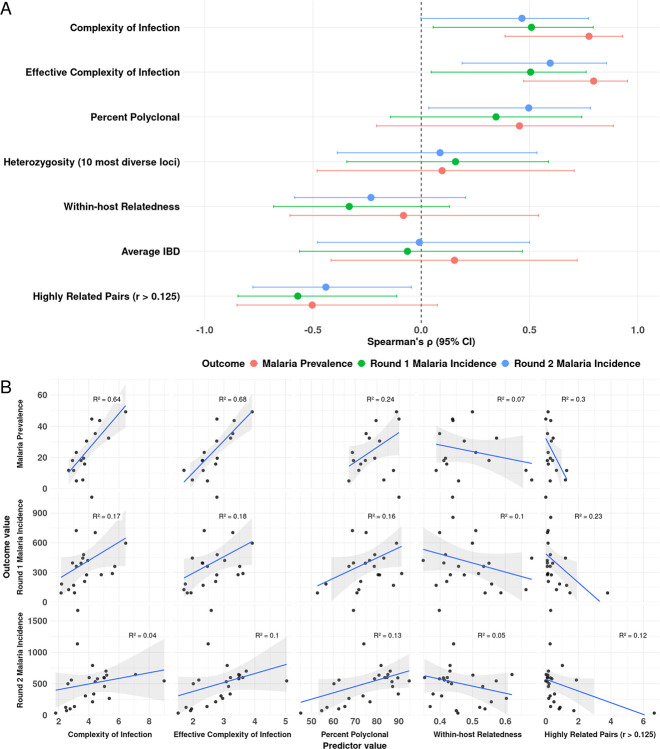
Correlation and association between parasite genetic diversity metrics and malaria transmission intensity across sites (all ages). (A) Spearman’s correlation coefficients (*ρ*) with 95% confidence intervals showing relationships between site-level epidemiologic metrics and parasite genetic metrics. (B) Linear regression analyses illustrating associations between epidemiologic metrics and selected genetic metrics. Each point represents a site-level estimate; blue lines denote fitted regression slopes with 95% confidence intervals, and *R*^*2*^ values indicate model fit.

To evaluate associations between parasite genetic metrics and transmission intensity (prevalence and incidence in the 3 months prior to sample collection), we first used the non-parametric Spearman’s rank correlation test (Figure 2A). **COI and eCOI** had the strongest positive correlation with parasite prevalence. **Percent polyclonal infections** and **heterozygosity** were also positively correlated with malaria prevalence, whereas **WHR** and the **proportion of highly related pairs (*r* > 0.125)** were negatively associated. Average IBD showed little association. Across both rounds of sample collection, incidence metrics showed weaker but directionally consistent associations. Similar patterns were observed when parasite genetic metrics were restricted to samples from participants < 15 years of age **(Supplementary Figure S5)**.

Linear regression findings were consistent with Spearman’s correlations; metrics of within-host parasite diversity were most strongly associated with malaria prevalence. **Effective complexity of infection (eCOI)** showed a robust positive relationship with prevalence (*R*^*2*^ = 0.68, *p* < 0.001, Figure 2B). **Complexity of infection (COI)** also exhibited a strong positive association with prevalence (*R*^*2*^ = 0.64, *p* < 0.01). The p**ercentage of polyclonal infections** was likewise positively associated with prevalence but to a lesser degree (*R*^*2*^ = 0.24, *p* > 0.05). In contrast, the **proportion of highly related pairs (*r* > 0.125)** was negatively associated with prevalence (*R*^*2*^ = 0.3, *p* < 0.05). Associations with incidence were more modest. Across both rounds**, percent highly related pairs (*r* > 0.125)** was negatively correlated with incidence, but the association was stronger in Round 1. While metrics of within-host diversity (COI, **eCOI**, and **percent polyclonal infections) were positively associated with incidence across both rounds, the associations were** weaker with incidence compared to prevalence. Restricting analyses to children under 15 years yielded similar effect sizes and directions **(Supplementary Figure S6)**, underscoring the robustness of these patterns across age strata.

### Estimating prevalence and incidence using genetic metrics

**Figure 3:**
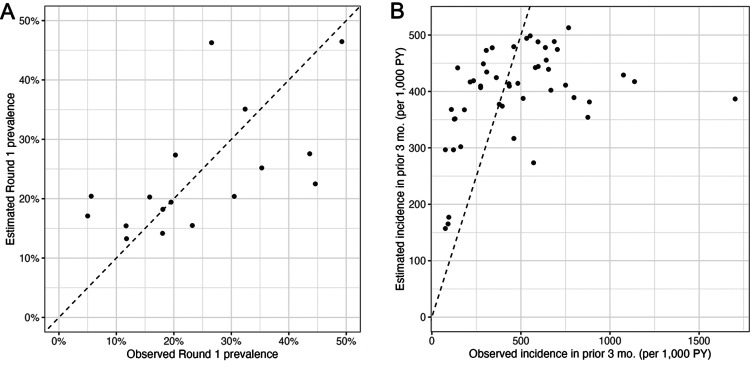
Observed versus estimated prevalence (A) and incidence in the prior 3 months (B) using the top performing models across 10 rounds of nested cross-validation. Prevalence was estimated using COI (*prevalence ~ COI*). Incidence was estimated using the mixed-effects negative binomial model described in the text (*incidence_count ~ percent polyclonal samples +% highly related samples + WHR + round + (1/site) + offset(log(exposure))*). Dotted line represents identity line.

Nested grouped cross-validation of regression models estimating prevalence repeatedly selected single-predictor models across 10 repetitions, indicating that adding additional genetic metrics did not improve performance. COI or eCOI was most frequently chosen, followed by percent polyclonal infections, consistent with the linear regression findings in Figure 2B. The mean RMSE was 0.12 ± 0.02, and the mean MAE was 0.10 ± 0.02, with a mean cross-validated correlation of r = 0.47 ± 0.12 between estimated and observed prevalence. When combined across folds and repetitions using Fisher’s pooled correlation the overall out-of-sample correlation between estimated and observed prevalence was r = 0.79 (95% CI 0.72–0.84), indicating that within-host diversity metrics explained approximately 62% of the variation in prevalence across sites. A scatterplot of estimated vs. observed prevalence for the top-performing model (COI as a single predictor) is shown in Figure 3A.

Nested grouped cross-validation of regression models estimating site-level incidence in the prior 3 months was performed separately for each round and then for the combined dataset. In analyses of individual rounds, model selection consistently favored single-predictor models based on the percentage of highly related samples. In contrast, when data from both rounds were combined into a single model that included round as a fixed effect, multi-predictor models incorporating both parasite diversity and relatedness metrics achieved the best out-of-sample performance. This pattern indicates that while the same molecular correlates of transmission intensity were evident across time points, models integrating multiple genetic metrics did a better job of capturing temporal variation. Across 10 repetitions, mean RMSE was 0.08 ± 0.002 and mean MAE was 0.06 ± 0.002, with a mean cross-validated correlation of *r* = 0.24 ± 0.10 between observed and predicted incidence per person-year. When results were combined across all folds and repetitions using Fisher’s pooled correlation, the overall out-of-sample correlation between estimated and observed incidence was *r* = 0.37 (95% CI 0.29–0.44), indicating that the model explained approximately 14% of the variance in site-level incidence. A scatterplot of estimated vs. observed incidence for the top performing model, which included percent polyclonal samples, within-host relatedness, and percent highly related samples as predictors, is shown in Figure 3B. Estimation was most accurate at < 200 cases per 1000 PY in the 3 months prior to sample collection and provided little information to distinguish between sites with incidence over 400. Model performance worsened when predicting incidence over longer prior intervals (6 or 12 instead of 3 months), indicating that smoothing incidence over longer intervals did not substantially improve predictive accuracy **(Supplemental Table 2)**.

## Discussion

This study genotyped samples from patients with malaria at health facilities across Uganda to evaluate the ability of parasite genetic metrics to estimate epidemiologic measures of transmission intensity. As expected, metrics of within-host diversity and parasite population diversity correlated positively with epidemiologic metrics, while metrics of within-host relatedness and parasite population relatedness were inversely correlated. Measures of within-host parasite diversity (COI and eCOI) were most strongly associated with malaria prevalence, explaining more than half the variation between sites. Relationships between parasite genetic metrics and incidence were weaker and varied more, with some metrics adding accuracy for seasonal changes in incidence. Together, these findings indicate that parasite diversity and relatedness metrics provide complementary information that capture broad differences in transmission intensity across sites, underscoring the potential utility of these metrics for estimating transmission intensity.

Within-host diversity metrics, in particular COI and eCOI, demonstrated strong accuracy for estimating prevalence, with a cross-validated pooled correlation of *r* = 0.79. In contrast, their performance for estimating incidence was substantially weaker, with models incorporating both diversity and relatedness metrics explaining only ~ 14% of the variance in site-level incidence. The weaker associations between genetic metrics and incidence compared to prevalence may reflect that these metrics are inherently less informative for estimating incidence compared to prevalence. While prevalence reflects cumulative infection pressure over time, incidence captures short-term dynamics influenced by climate factors such as rainfall and temperature, health-seeking behavior, and diagnostic completeness (1, 26, 28). Short-term changes in incidence may outpace the rate at which the parasite population changes, reducing the sensitivity of genetic metrics to short-term transmission dynamics.

Inaccurate measurement or temporal and spatial variability of the incidence metric may also contribute to the relatively poor performance of the incidence models. Although incidence data were collected from sentinel surveillance sites with enhanced quality control, a few sites reported unusually high incidence estimates, possibly due to errors in the population denominators used to calculate incidence. These outliers likely had a disproportionate effect on the associations between parasite genetic metrics and incidence due to the limited sample size and may explain the overall underestimation of incidence observed in the model. In addition, incidence metrics at these sites were calculated by dividing the number of cases coming from the “target area” divided by the population of the target area, while the samples were collected from any patient attending the health facility, regardless of whether they lived in the target area. Unfortunately, too few samples were collected to limit the analysis to those collected only from patients in the target area.

To our knowledge, only one other study has attempted to directly estimate transmission intensity from parasite genetic data. In 2024, Wong et al. used parasite genetic metrics derived from 24-SNP barcode data collected from 16 sites across Senegal to estimate annual malaria incidence by site and year, comparing estimates from Poisson generalized linear mixed-effects models against incidence data reported by Senegal’s NMCP. We observed weaker relationships between within-host diversity metrics such as percent polyclonal infections and malaria incidence compared to their findings. Several methodological differences likely contribute. First, outcome definition and geographic scale differed substantially between the studies. Wong et al. used NMCP annual incidence at the district level in Senegal, which was sometimes aggregated to region-level when clinic-specific data were unavailable. In contrast, we used incidence data specific to each site and over a shorter timeframe, which may have increased variability. However, increasing the time period of the incidence outcome did not improve our ability to estimate incidence. Second, although both studies evaluated performance by holding out whole sites, Wong et al. used a single-level leave-one-site-out approach, whereas we implemented *nested* site-grouped cross-validation, which separates model selection from evaluation and offers stronger protection against overfitting. Third, our study spanned many more sites in the intermediate to high incidence range, which our data suggest may be more difficult to distinguish using molecular metrics. Finally, substantially different genotyping methods were used (SNP barcoding versus polyallelic microhaplotypes) and therefore different tools were used to estimate molecular metrics (15). Taken together, these factors may account for the lower explanatory power of our incidence models relative to those reported by Wong et al.

Between-host relatedness, measured as the site-level percentage of highly related samples by IBD, showed a negative correlation with transmission intensity and was the predictor most consistently selected in both single-round and combined-round incidence models. This pattern is consistent with population genetic theory that lower-transmission settings experience stronger bottlenecks that generate more closely related parasite lineages. Similar inverse relationships between genetic relatedness and transmission intensity have been reported in prior studies that used SNP barcoding techniques to estimate the fraction of non-unique monogenomic clones or identical barcode haplotypes across seasons (15, 35, 36). In high transmission settings, SNP barcoding approaches lose discriminatory power because high levels of multiclonality can mask underlying haplotypes and reduce the discriminatory power of barcode SNPs (37, 38). An advance of this study relative to the SNP barcoding approach is the ability to estimate IBD from polyallelic microhaplotype data using Dcifer (20), rather than limiting relatedness analyses to exact barcode matches, which may have improved our ability to detect differences in relatedness across sites.

This study benefited from several notable strengths. A large and geographically diverse set of sites across Uganda was included, spanning a wide range of transmission intensities and enabling both spatial and temporal comparisons. The use of a highly sensitive microhaplotype panel, together with analytical tools such as MOIRE and Dcifer that leverage the full information content of polyallelic loci, address key limitations of traditional SNP barcoding (20, 22, 37). The analytical framework presented incorporated rigorous cross-validation procedures to assess out-of-sample performance, reducing the likelihood of overfitting. To our knowledge, no prior study has paired health-facility based genotyping data with both prevalence and incidence estimates across multiple locations, allowing for a more direct comparison of parasite genetic diversity and epidemiologic indicators than previously performed.

Despite these strengths, several limitations should be acknowledged. Although the study included sites across a broad range of transmission intensities, the majority were characterized by high transmission, which may have limited the ability to detect relationships that emerge more clearly in lower-transmission settings or across a broader range of transmission intensity. Samples were collected from all age groups; infections in school-aged children, who have developed anti-disease immunity but continue to have high parasite density infections and contribute disproportionately to ongoing transmission, may contain additional information (39). However, we did not find evidence of this when we limited our parasite genetic analysis to samples in children less than 15 years of age. In addition, samples collected from symptomatic patients presenting to health facilities may represent infections that are dominated by new clones and may not fully capture the relationship between within-host parasite diversity and transmission intensity. This limitation will be addressed in future studies incorporating genotyping from cross-sectional sampling. Only 17 sites with genetic data had prevalence data available, and prevalence estimates were limited to a single time point, which constrained the ability to examine temporal trends. Finally, some of the observed uncertainty in relationships between genetic measures and incidence may reflect inaccuracies or inconsistencies in incidence estimates or imperfect alignment between the populations contributing clinical cases and those used for surveillance-based incidence calculations. These considerations indicate that additional work integrating harmonized epidemiologic and genomic sampling frames will be valuable for future studies.

## Conclusion

In summary, parasite genetic metrics derived from high-resolution microhaplotype data consistently differentiated higher- from lower-transmission sites and accurately captured spatial patterns observed in prevalence. Measures of within-host diversity, particularly COI and eCOI, showed strong and consistent associations with parasite prevalence and demonstrated substantial predictive accuracy. A combination of within-host diversity and between-host relatedness metrics was most informative for predicting incidence, but showed relatively poor predictive performance. Together, these results indicate that microhaplotype-based measures of parasite diversity and relatedness capture broad spatial and temporal gradients in transmission intensity, although their capacity to meaningfully estimate incidence remains unclear. Future work to better assess the added value of parasite genetic metrics to estimate transmission should incorporate community-based sampling to capture asymptomatic infections, expand sampling in lower-transmission settings, and pair genotyping with harmonized epidemiologic data collection to further refine molecular tools for transmission estimation. Regional standardization of genetic metrics and analytic pipelines, coupled with cost-effectiveness evaluations, will be essential for sustainable implementation within NMCP frameworks across sub-Saharan Africa (29, 40).

## Supplementary Material

Supplementary Files

This is a list of supplementary files associated with this preprint. Click to download.

- Supplementarydatav5.docx

## Figures and Tables

**Table 1.** Comparison of Participant Characteristics Between Round 1 (January – March) and Round 2 (July – September)

Characteristic	Round 1 (Jan – Mar) Median [Q1–Q3] or %	Round 2 (Jul – Sept) Median [Q1–Q3] or %
Sample size	1510	2053
Number of study sites*	26	26
Number of sites with prevalence data	17	NA
Number of sites with incidence data	23	25
Sample size per site	57 [45–72]	78 [68–92]
Parasitemia (parasites/μL)	9939 [2115–38756]	13786 [3678–53737]
Age (years)	10 [4 – 15]	8 [3 – 14]
Age categories		
Age less than 5 years	26.7%	33.9%
Age 5–15 years	44.6%	44.3%
Age over 15 years	28.7%	21.8%

* Only 23 out of the 26 sites are the same for both Round 1 and Round 2. Three sites (Alebtong, Kiyunga, and Bikurungu) are only in round 1 while 3 sites (Awach, Ayipe, and Diima) are only in round 2.

## Data Availability

The IMMRSE-U study datasets are available in the study database and will be publicly accessible upon publication. Additional data is provided within the manuscript or supplementary information files.
